# Effect of cold storage on Carbofuran dissipation in Cabbages (Brassica Oleracea)

**DOI:** 10.1186/s13065-022-00867-1

**Published:** 2022-10-19

**Authors:** Maeena Naman Shafiee, F. A. Masoodi, Sajad Mohd Wani, Tehmeena Ahad

**Affiliations:** 1 Department of Food Science and Technology, University of Kashmir, Srinagar, Kashmir India; 2 Department of Food Science and Technology, Sher-I-Kashmir University of Agricultural Sciences, Srinagar, Kashmir India

**Keywords:** Cabbage, Carbofuran, Half-life, Dissipation

## Abstract

Cabbage being a highly imported product is often subjected to long term cold storage to maintain product quality and in order to retain its freshness it is kept in cold chambers for a long time. Being highly infested by pests during storage, cabbages are often treated with insecticides having active ingredients such as carbofuran in them. Also, large number of malpractices have come into the notice of the regulatory bodies where the growers use last minute Carbofuran dips on the cabbage heads to improve the shine and lustre of the foliage for better marketability .Therefore, the study was conducted to monitor the effect of cold storage on degradation pattern of Carbofuran on Cabbage during storage in Kashmir valley. The level of carbofuran residue decreased with an increase in storage period Dissipation pattern was studied for three application rates of Carbofuran by dipping the samples in T1 (First dose) 3g/1000mL, T2 (Second dose) 2g/1000mL, T3 (Third dose) 1g/1000mL. The initial control deposit was found to be 0.05mg/kg for T1, 0.03mg/kg for T2, 0.01mg/kg for T3 on Cabbage. The residues were dissipated to about 0.02mg/kg in T1, 0.01mg/kg in T2 and ND in T3 after 60 days of application. The waiting period after proper risk assessment was calculated and was found to be 30 days for almost all application rates.

## Introduction

Cabbage (Brassica Olerecea) is being used for numerous medicinal properties since ages. It has number of anti-inflammatory and anti-carcinogenic properties. This is because the presence of indole photochemical which work up the female hormones of oestrogens and produce cell changes that prevent colon cancer (Allen 2009). Cabbage contains number of nutrients including certain essential vitamins and minerals like calcium and Vitamin C (Norman 1992). India is the second largest producer in the world of cabbage after china (IHDB (Indian horticultural data base) 2009). In Kashmir the total area under cultivation for cabbage is 249 hectares with an annual production of 7323 tonnes (Directorate of Statistics and Economics, 2016).

Despite its high medicinal value and huge production, cabbage production in Kashmir valley has numerous constraints, one of them being pest infestation that reduces the yield of crop. This crop is under constant threat of pest infestation from insect pests like sucking and defoliating insects’ right from germination to the harvesting stage. This pest infestation limits the yield, forcing the farmers to use pesticides on regular basis to improve the crop quality (Legwaila et al. 2014). In India the most common pest that are reported to have caused maximum damage are diamond back moth (Plutella xylostella Linneaus) (Younas et al. 2004). Hence numerous pesticides are thereby recommended to be used for the control of this pest infestation and Carbofuran (2,2-Dimethyl-3H-1-benzofuran-7-yl) N-methylcarbamate) is one of them. It is the most toxic systemic insecticide for the control of the pests in cabbage. Carbofuran is a systemic insecticide, the mode of action for the same includes endocrine disruption with probable reproduction and development of intoxicants. It also leads to alterations in the concentration of hormones (IUPAC). Carbofuran and its metabolite, 3-hydroxycarbofuran, irreversibly inhibit acetycholinesterase (AChE) which leads to the continuous action of the hydrolysed neurotransmitter on its postsynaptic receptors (Jongeneelen et al. 2013).

In Kashmir the growers use Carbofuran, just before the harvest to prevent pest defoliate from diamond back moth. Therefore, this practise may leave residues which can be extremely hazardous for human health. The presence of this residue is based on numerous factors like dosage applied, waiting period between post-harvest and pre culinary, crop variety and agro climatic conditions. It must also be noted that Cabbages are often stored in cold storage for a long span of time before they are exported to various places in the world. So far there is no information available on the dissipation pattern of Carbofuran in cold storage and how much waiting period must be given to the vegetable before consumption. The present study was therefore conducted to investigate the dissipation pattern of Carbofuran at different periods of cold storage.

## Materials and methods

### Chemicals and reagents

The Carbofuran reference standard (purity 99.5%) was purchased from Sigma-Aldrich, USA. A Carbofuran market formulation (Carbo–G) obtained from M/S Premium sales agency Srinagar was used for Dipping. Acetonitrile (high-performance liquid chromatography (HPLC) grade) and acetic acid were purchased from HIMEDIA, India and sorbents for QuEChERS (Quick, Easy, Cheap, Effective, Rugged and Safe) analysis like primary secondary amines (PSA), anhydrous magnesium Sulphate, sodium acetate were all purchased from Sigma-Aldrich, USA. All the reagents and sorbents were kept at 4 ^0^C or as directed by the manufacturer. Blanks samples were also run to test the reagents for purity.

### Preparation of standard solution

The stock standard solution of Carbofuran (1mg. mL^− 1^) was prepared in HPLC grade hexane. The standard solutions were required for constructing a calibration curve (20, 50,100,200 ppb) from serial dilution with hexane. All standard solutions were stored at refrigerated conditions (4 ^0^C) before use.

### GC-MS analysis (gas chromatography-mass spectroscopy)

The detection of Carbofuran was done by gas chromatography-mass spectroscopy (Agilent 6890N) equipped with flame photometric detector (NPD AND NPD^+^). The column used for separation was RTX-5MSX-0.25mm, carrier gas used for elution was Helium at 30cm.min at a temperature of 80^o^ Celsius- on hold for 0min and then the temperature was increased at 20^o^celcius for 4.75min and gradually increased by gradient up to 290 ^o^C. This was then kept on hold for 4.75min. The injection was Split less at 2 micro litres. Mass Spectroscopy was done at the capillary voltage of 3KV where ionisation mode was kept positive with a temperature of 275^o^C (desolvation). The Gas temperature was regulated at 275 lit/hr. The total run time was that of 30min. Data compilation and analysis was performed by Mass hunter Software under MRM mode. Simple probe homogeniser WiseTis® was used for homogenising the sample. For sample preparation eppendorf®5810R centrifuge was used.

### Sample preparation

This study was carried out on Cabbage (var. Pride of India) at University of Kashmir, Hazratbal during the autumn harvest season of 2020. The samples collected were from the local market base and were first tested for interferences by GC/MS (Matrix effect). Interferences being found as negative, the samples were washed and dipped in the commercial formulation of Cabbage. The formulation was diluted in the distilled water at the concentration of 3g/1000mlL (T1), 2g/1000mL (T2), and 1g/1000mL (T3). The samples were allowed to rest in the formulation for 2h. The samples for 0 weeks (as soon as the solution dried up) were collected and analysed while as rest of the samples were stored at 4 ^0^C for 2 months. The change in the residual concentration was monitored at regular intervals of 0, 1, 2 months.

### Processing of samples

Cabbage Samples were chopped on a simple chopping board and blended in an electric blender with 1000rpm. After grinding, sample was homogenised in a homogenizer (Witeg, Germany). Pesticide residues were then extracted using QuEChERS method (AOAC.2007) with dispersive clean-up. About 15 mL of homogenised sample was used during comminution and mixed with 6g of anhydrous MgSO_4_ in a 15 mL centrifuge tube. 1.5g of sodium acetate was used as a buffer and mixed with 15 mL of 1% acetic acid of acetonitrile. The tubes were tightly closed and vigorously shaken for 1min on a vortex mixer for 2min. The samples were centrifuges at 1500 rcf for 1min.

For d-SPE, 1–8 ml of supernatant was added to a centrifuge tube containing 150mg anhydrous MgSO4 along with 50mg of PSA. The tubes were shaken well for 2min on a vortex mixer and then centrifuged for 1500 rcf for 1min. About 1 ml of supernatant was collected using a micropipette and transferred to auto sampler vial of GC/MS.

### Degradation kinetics

The degradation kinetics of the pesticide residue in Cabbage was obtained by residue concentration versus time graph. The degradation trend followed was that of first order rate equation. This was validated by graphical representation of lnC against time. The degradation rate constant was calculated by Ct = Coe − kt, where Ct is the concentration of the pesticide residue at time t, Co is the initial concentration of the sample, and k is the rate constant in days − 1.

Mean lifetime τ, half-life (t_1/2_), Decay constant λ were calculated using respective chemical equations and formulas as given under

$$\begin{array}{c} N\left( t \right) = NO(1/2)t/t1/2,N\left( t \right)\\ = NOe - t/\tau ,N\left( t \right) = NOe - \lambda t\end{array}$$

Where No is the initial quantity (before storage), N(t) is the residual quantity at regular monitoring intervals after time t, τ is the Mean lifetime, t_1/2_ is the half-life, λ is the decay constant. Half -life was used to calculate the k value and In 2/k for each experiment. (Wang et al. 2007)

### Risk assessment

The risk assessment with respect to Carbofuran dissipation in storage was calculated by evaluating dietary exposure with maximum permissible intake (MPI). The prescribed ADI (acceptable daily intake) for Carbofuran is 0.002mg/kg per body weight per day (WHO,2003). Considering the average weight of an individual as 55kg, the MPI was found to be.

0.55mg/kg. On the basis of average per capita consumption of fruits and vegetables, an average of 80g is consumed by the consumer per day (Mukherjee et al. 2021).

## Results and discussion

The limit of detection was found to be 0.01mg/kg and the limit of quantification was found to be 0.025mg/kg for Carbofuran in Cabbage. Method validations was carried out for cabbage samples by fortifying them with 20, 50,100,200 ppb in triplicate and were extracted by the same method as described above. The recovery percentage was found to be commendable in the range of 81.43 to 91.97%.

Relative standard deviation was calculated in % RSD and ranged between 0.73 and 1.22% (Table 1).

**Table 1:** Recovery percentage of Carbofuran from Cabbage

Fortification levels (ppb)	Replicate Recovery (%)	Average Recovery (%)	Relative Standard Deviation (%)
20	83.45 82.46 84.5	83.47 ± 1.02	1.22
50	81.60 82.3 80.4	81.43 ± 0.96	1.18
100	92.43 91.2 92.3	91.97 ± 0.67	0.73
200	85.65 85.4 84.3	85.1 ± 0.71	0.84

**Fig. 1:**
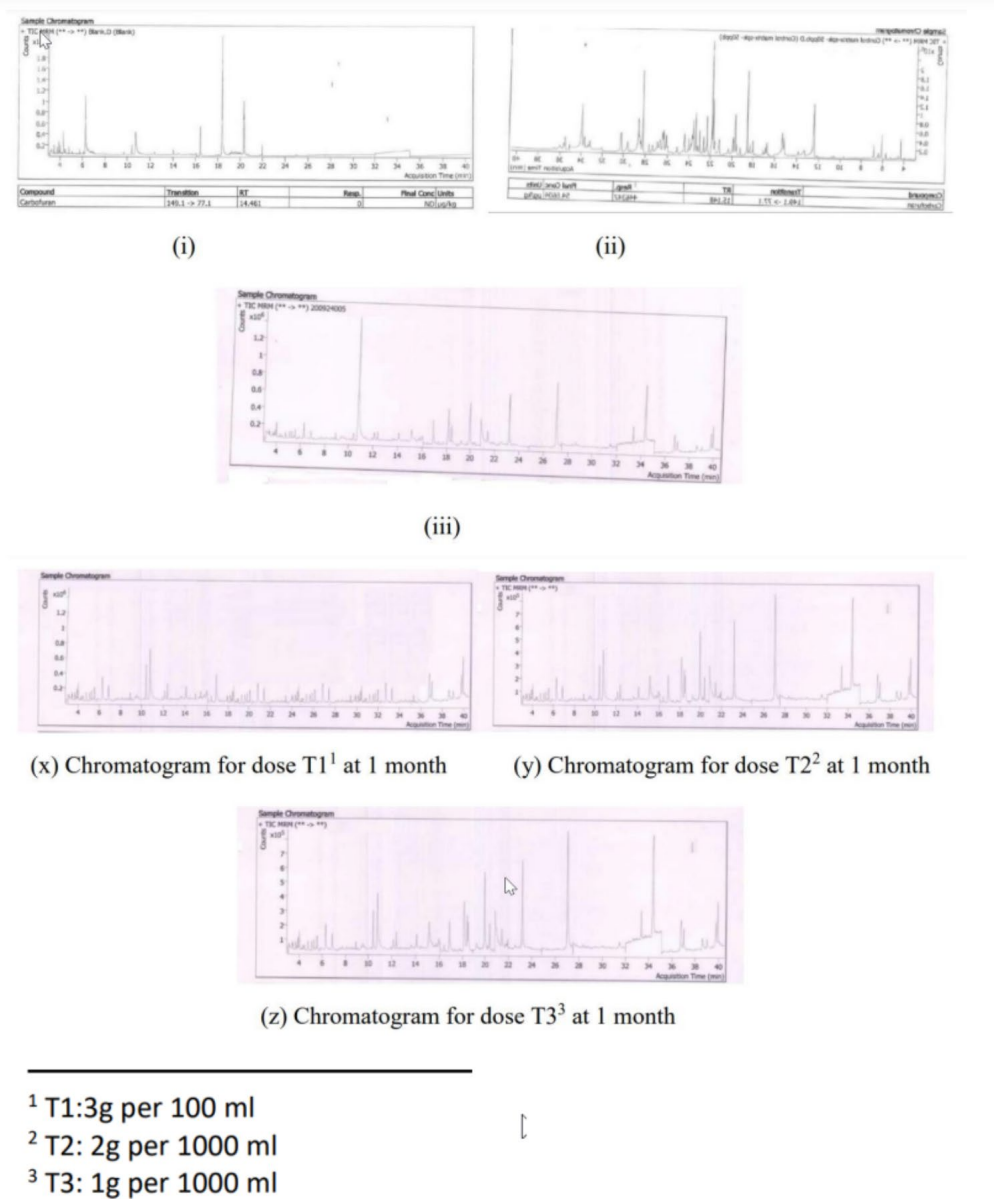
**Chromatogram of Carbofuran (i) Blank (ii) Standard (iii) Samples**

No correction factors were applied. The chromatograms for Blank, Fortified Samples and Standard are as below Fig. 1:

The dissipation trend for Carbofuran is presented in Table 2. Average initial deposit of the pesticide was found to be 0.05mg/kg, 0.03mg/kg, 0.01mg/kg respectively in T1, T2, T3 doses. After 30 days of storage at 4^o^ Celsius the residues degraded substantially and reached 0.02mg/kg, 0.01mg/kg, 0.00mg/kg in three doses respectively. On 60th day the residues were found to be negligible in all the three doses with a percentage decrease of 99.0%, 99.9.19%, 100% respectively.

**Fig. 2:**
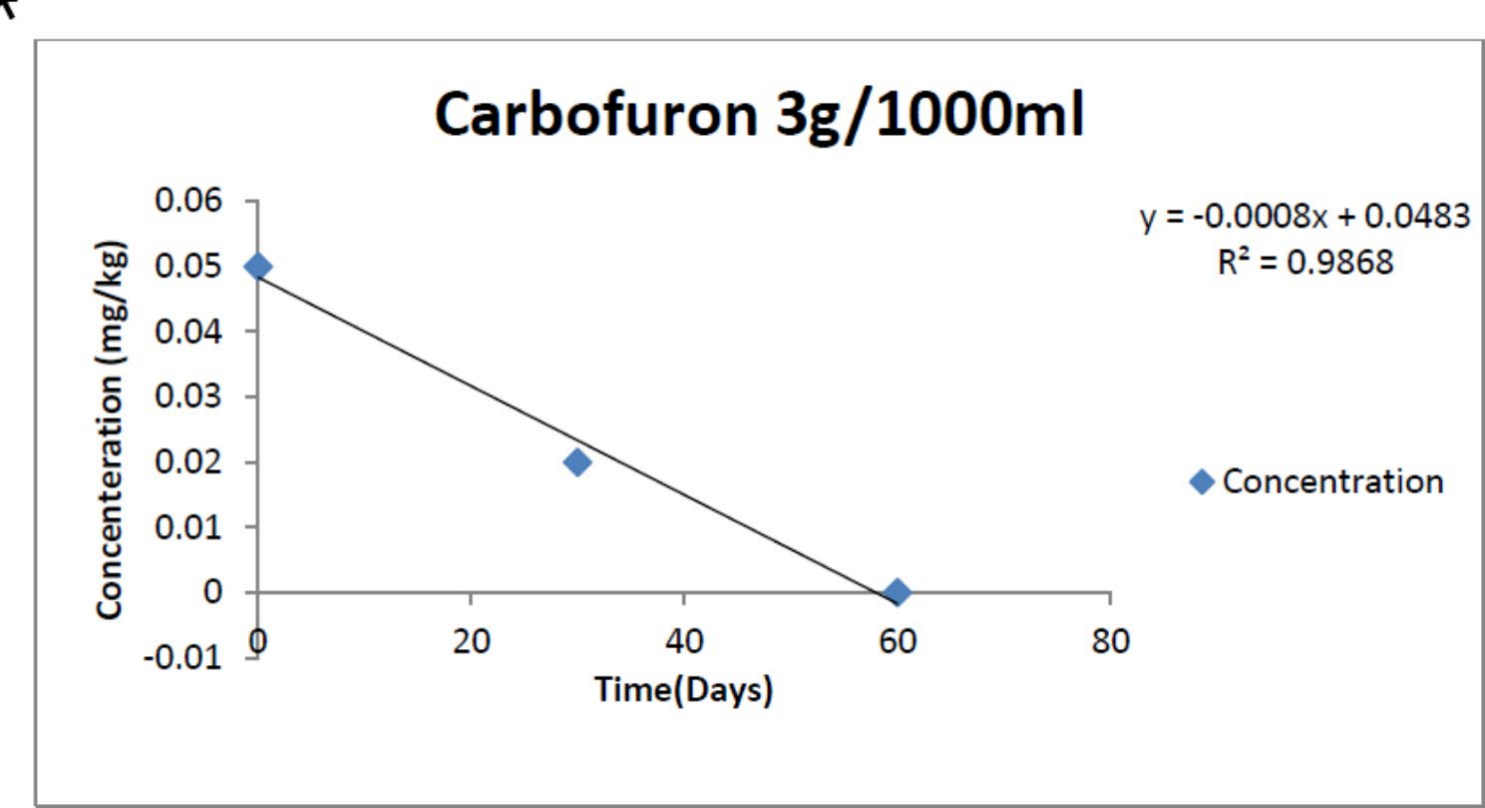
Graph of residue dissipation Vs Time following first order kinetics for dose T1 (T1: 3g per 1000 ml)

**Fig. 3:**
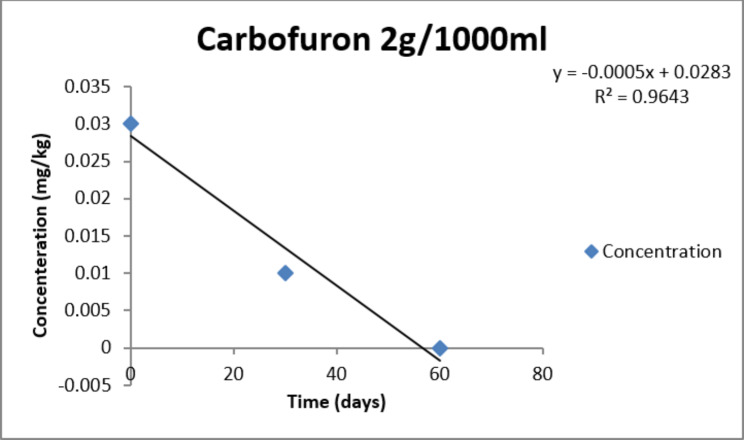
Graph of residue dissipation Vs. Time following first order kinetics for dose T2(T2: 2g per 1000 ml)

After 60 days the percentage reduction of the residues was found to be 99.0%, 99.9.19%, 100% respectively in three doses respectively. Residual concentration versus time relationship can be seen in Figs. 2 and 3.

The trend in the reduction of the pesticide residue analysis can be observed by the exponential decrease in the residual concentrations as it follows the first order rate of kinetics. The kinetic equation followed is that of Ct = Coe − kt Where concentration.

(C) and Time (t) are found to be as in Table 2. Data for all the three doses was subjected to statistical analysis and calculation of half-life, decay percentage and mean lifetime were calculated as in Tables 3 and 4. Therefore, for the safety of consumers the waiting period was found to be 30 days for Carbofuran treated Cabbage under storage in Kashmir valley.

**Table 2:** Dissipation trend of Carbofuran in Cabbage

Carbofuran dissipation for dose T_1_ following Ct *= Coe − kt*								
**Ct (days)**	**k**	**T**	**c**_**i**_	**Ln c**_**i**_	**Ln c**_**t**_	**Final Values**	**Real Lab Values (mg/kg)**	**Percentage decrease**
0	0.03	0	0.05	-2.995	-2.99	0.05	0.05	0
30	0.03	30	0.05	-2.99	-3.89	0.020	0.02	93.77%
60	0.03	60	0.02	-3.9120	-5.712	0.003	ND	ND

Half Life, t^1/2^: 22.69 (days)

Mean lifetime, τ: 32.74 (days).

Decay constant, λ: 0.030.

MRL: 0.1mg/kg.

Waiting Period: 30 (days).

Co-relation Coefficient: 0.98.

**Table 3:** Dissipation trend in Carbofuran in Cabbage

Carbofuran dissipation for dose T2 following *C*_*t*_*=Coe − kt*								
**ct (days)**	**k**	**t**	**ci**	**Ln** **ci**	**Ln ct**	**Final concentration**	**Real Lab Values (mg/kg)**	**Percentage decrease**
0	0.03	0	0.03	-3.50	-3.50	0.03	0.03	0
30	0.03	30	0.03	-3.56	-4.40	0.012	0.01	66.67%
60	0.03	60	0.01	-4.605	-6.40	0.0016	ND	ND

Half Life, t^1/2^: 18.92 (days)

Mean lifetime, τ: 27.30 (days).

Decay constant, λ: 0.036.

MRL: 0.1mg/kg.

Waiting Period: 30 (days).

Co-relation Coefficient: 0.96.

**Table 4:** Dissipation trend of Carbofuran in cabbage

Carbofuran dissipation for dose T3 following ***C***_***t***_***=Coe − kt***								
**ct (days)**	**k**	**t**	**ci**	**Ln** **ci**	**Ln ct**	**Final concentration**	**Real Lab Values (mg/kg)**	**Percentage decrease**
0	0	0	0.01	-4.605	-4.60	0.01	0.01	0
30	0	30	0.01	-4.605	-4.60	0.01	ND	0.00%
60	0	60	0.01	-4.605	-4.60	0.01	ND	ND

A similar study was carried out in kale and brinjal and half-life for leaves was also reported to be less than the fruits. The half-life of kale was found to be 2.54 days while as in brinjal leaves and fruits it was longer that is 3.22 and 10.33 days, respectively (Sim et al. 2019).

It was also observed by Cabras et al. 1988 in lettuce that very low concentration of Carbofuran and its metabolites were found with reduced half-life when used in lettuce.

## Statistical analysis

### ANOVA analysis for carbofuran dissipation pattern

One way ANOVA was conducted to analyse whether there is any significant change in the dissipation pattern with the difference in the varying concentration. The hypothesis of the study included *H*_*0*_ *= There is no change in the dissipation trend with varying concentration & H*_*1*_ *= There is significant change in the dissipation trend with varying concentration* Results of the same are summarised in Tables 5 and 6.

**Table 5:** Results for ANOVA

Groups	N	Mean	Std. Dev.	Std. Error
Dose T1	3	0.0233	0.0252	0.0145
Dose T2	3	0.0133	0.0153	0.0088
Dose T3	3	0.0033	0.0058	0.0033

**Table 6:** ANOVA Summary

Source	Degrees of Freedom	Sum of Squares	Mean Square	F-Stat	P-Value
	DF	SS	MS		
Between Groups	2	0.0006	0.0003	0.9969	0.4228
Within Groups	6	0.0018	0.0003		
Total:	8	0.0024			

Null hypothesis is accepted as P value (0.097) is larger than 0.05 and F stat value is smaller than F critical (5.41). Acceptance of Null Hypothesis thereby states that whatever the concentration of the dose applied on the produce, the dissipation trend will remain the same i.e., decreasing with the increase in time in accordance with the first order of chemical kinetics.

## Risk assessment

The theoretical maximum residue contribution (TMRC) was found to be as stated in Tables 7 and 8.

**Table 7:** Risk assessment for Carbofuran dose T1

Time	Pesticide ADI (Mg/kg)	Avg. wt. of person kg	MPI (Mg/kg/person/day)	Avg. Residue (mg/kg)	Avg. Consumption (g)		TMRC (mg/kg)
0	0.002	55	0.11	0.05		0.08	0.004
30	0.002	55	0.11	0.02		0.08	0.0016
60	0.002	55	0.11	0		0.08	0

**Table 8:** Risk assessment for Carbofuran dose T2

Time	Pesticide ADI (Mg/kg)	Average wt. of person (kg)	MPI (Mg/kg/ person/day)	Average Residue (mg/kg)	Average Consumption (g)	TMRC (mg/kg)
0	0.002	55	0.11	0.03	0.08	0.0024
30	0.002	55	0.11	0.01	0.08	0.0008
60	0.002	55	0.11	0	0.08	0

Since the TMRC Values decrease significantly after 60 days and are in below limit of detection after 60 days the waiting period was thus found to be 30 days. This therefore imposes minimum health risk to the consumers if consumed after the waiting period of 30 days.

## Conclusion

QuEChERS method of extraction was used for extraction of Carbofuran in the Cabbage samples. The limit of detection was found to be 0.01mg/kg and the limit of quantification was found to be 0.025mg/kg for Carbofuran in Cabbages. The recovery percentage was found to be commendable in the range of 81.43 to 91.97%. After 60 days the percentage reduction of the residues was found to be 99.0%, 99.9.19%, 100% in three doses respectively. The Half-life was found to be 30 days for all the three doses respectively. The trend in the reduction of the pesticide residue analysis can be observed by the exponential decrease in the residual concentrations as it followed the first order rate of kinetics. Since the TMRC Values decrease significantly after 60 days and are in below limit of detection after 30 days the waiting period was thus found to be 30 days. This therefore imposes minimum health risk to the consumers if consumed after the waiting period of 30 days.

## Data Availability

The datasets generated and/or analysed during the current study are not publicly available due to privacy of data but are available from the corresponding author on reasonable request.
